# Predictive value of geriatric nutritional risk indexes for hospital readmission and mortality in older patients

**DOI:** 10.1007/s40520-024-02899-0

**Published:** 2024-12-27

**Authors:** Yajun Chen, Jin Wei, Ming Zhang, Dongping Xu, Yuheng Lang, Yumei Qi

**Affiliations:** 1 Department of Nutrition, National Clinical Research Center for Cancer. Tianjin’s Clinical Research Center for Cancer. Key Laboratory of Cancer Prevention and Therapy, Tianjin Medical University Cancer Institute & Hospital, 300060 Tianjin, China; 2 Tianjin Key Laboratory of Extracorporeal Life Support for Critical Diseases, Artificial Cell Engineering Technology Research Center, The Third Central Hospital of Tianjin, Tianjin, China; 3 Tianjin Institute of Hepatobiliary Disease, Tianjin, China

**Keywords:** Geriatric nutritional risk index, Older inpatients, Clinical outcomes, Hospital readmission, Mortality rates

## Abstract

The Geriatric Nutritional Risk Index (GNRI) plays a crucial role in assessing nutritional status and predicting clinical outcomes in older patients. This study explores the GNRI as a prognostic tool for clinical outcomes in older inpatients. Spanning from August 2013 to December 2020, the research involved 573 older patients at Tianjin Third Central Hospital, China. The study assessed the association of GNRI with 6-month hospital readmission and 3-year all-cause mortality rates. Our findings reveal that higher GNRI scores significantly correlate with reduced hospital readmissions and mortality, underscoring the utility of GNRI in predicting clinical outcomes and guiding interventions in geriatric care. The study highlights the potential of integrating GNRI assessments into routine clinical evaluations to enhance patient care and optimize resource utilization in healthcare settings.

## Introduction

The Geriatric Nutritional Risk Index (GNRI) plays a crucial role in assessing nutritional status and predicting clinical outcomes in older patients across various medical conditions. Studies have shown that a lower GNRI is associated with adverse outcomes such as postoperative delirium after cardiac surgery [1], disease activity in rheumatoid arthritis patients [2], muscle volume loss in chronic liver disease patients with hepatocellular carcinoma [3], and mortality in heart failure patients [4] and incident hemodialysis patients [5]. GNRI is a simple yet effective tool that can help identify nutritional risk, predict mortality, and guide clinical interventions in older populations, making it a valuable asset in geriatric care and various medical specialties.

The GNRI holds significance as a predictive tool for various clinical outcomes. Research indicates that GNRI is associated with disease activity in older rheumatoid arthritis patients [2], serves as a prognostic indicator in metastatic urothelial carcinoma patients undergoing chemotherapy [6], and predicts outcomes in stomach cancer surgery patients, including overall survival, cancer-specific survival, and post-operative complications [7]. Moreover, GNRI has been studied in the context of machine learning pipelines for clinical data classification, emphasizing the importance of fairness and bias mitigation strategies in AI systems [8, 9]. Overall, GNRI emerges as a valuable tool for predicting hospital stay, readmission rates, and overall survival across various medical conditions, highlighting its potential in enhancing patient care and treatment outcomes.

The primary aim of the study was to investigate the relationship between the GNRI and various clinical outcomes in older inpatients. This included assessing the predictive value of GNRI for 6-month hospital readmission and 3-year all-cause mortality rate. The proactive use of GNRI as a screening tool in clinical settings enables healthcare providers to intervene early, potentially reducing hospital stays and enhancing the quality of life for older patients.

## Subjects and methods

### Patients

This study is a retrospective analysis of 573 patients admitted to the Department of General Medicine of Tianjin Third Central Hospital, China between August 2013 and December 2020. Inclusion criteria: (1) patients aged ≥ 60 years old; (2) voluntary to participate in this study. Exclusion criteria: (1) patients with mental illness or cognitive communication disorders; (2) metal stents or pacemakers placed in the body; (3) patients with acute diseases. A total of 573 older inpatients were included. Clinical data were collected from both inpatient and outpatient medical records. This study was approved by the Ethics Committee of The Third Central Hospital of Tianjin (IRB2020-012-01). All patients or their guardians provided informed consent prior to enrollment in this study.

### Data collection

#### General Information

General information included gender, age, height, weight, and body mass index (BMI). BMI = body mass (kg)/height^2^ (m^2^).

#### Determination of body composition

The InBody S10 produced by Korea Biospace Inbody Body Composition Analyzer Co., Ltd. was used for measurement. After the measurement was completed according to the operating standard, the body composition indexes were recorded, including intracellular water (ICW), extracellular water (ECW), total body water (TBW), body cell mass (BCM), skeletal muscle (SM), protein (PRO), body fat (BF), fat-free mass (FFM), visceral fat area (VFA).

#### Laboratory indexes

Laboratory indexes included total protein (TP), serum albumin (ALB), hemoglobin (Hb), white blood cell (WBC), red blood cell (RBC), lymphocyte count (LYM); Serum rapid reaction protein: prealbumin (PA), transferrin (TRF), retinol-binding protein (RBP), fibronectin (FN). Blood samples were taken on an empty stomach in the morning.

### Evaluation and judgment criteria of GNRI

GNRI = 1.489 × Alb + 41.7 × (actual weight/ideal weight). For patients with difficulty in standing up and unable to measure height, height can be estimated by measuring knee height. Male height = 2.02 × knee height (cm) − 0.04 × age + 64.19; female height = 1.83 × knee height (cm) -0.24 × age + 84.88. The GNRI was determined by calculating the average value based on the results of three repeated measurements. NRI nutrition assessment grade criteria: patients with GNRI > 98 were assigned into high GNRI group while GNRI ≤ 98 were assigned into low GNRI group. If the actual weight was greater than the ideal weight, the ratio of actual weight to ideal weight was calculated as 1.

### Clinical outcome indicators

Length of stay (LOS) and hospital costs (HC) were used as clinical outcome indicators.

### Statistical analysis

Data were analyzed by SPSS 22.0 software. The data were tested for normal distribution. Quantitative data conforming to normal distribution were expressed as means ± standard deviation (SD). Grading data and qualitative data were expressed by frequencies and percentages. One-way analysis of variance was used for comparison between groups of quantitative data with normal distribution and homogeneity of variance after Ln conversion. The comparison of categorial data was performed by Chi-square test. Univariate logistic regression was performed to identify factors significantly associated with 6-month hospital readmission. Each variable was analyzed independently to determine its unadjusted effect on the outcomes. Subsequent to the univariate analysis, significant variables (*p* < 0.05) were included in a multivariate logistic regression model to adjust for potential confounders. The ROC analysis was conducted to evaluate the sensitivity and specificity of the GNRI as a predictive tool for hospital readmission. Overall Survival curves were plotted using the Kaplan–Meier method and compared using the generalized log-rank test. *p* < 0.05 was considered statistically significant.

## Results

### Association of GNRI and patient characteristics

Table 1 presents a comparative analysis of older inpatients categorized into High GNRI (*n* = 241) and Low GNRI (*n* = 332) group based on their NRI scores. Key variables assessed include age, sex, diseases, body metrics, and laboratory test results. Weight and BMI showed statistically significant differences (*p* = 0.004 and *p* = 0.026, respectively). Noteworthy laboratory differences include total protein and albumin levels, which were significantly higher in the High GNRI group compared to the Low GNRI group (*p* = 0.002 and *p* = 0.019, respectively). These findings suggest that higher GNRI values are associated with better nutritional and physiological profiles, which may influence clinical outcomes in these patients.

**Table 1:** Association of GNRI and patient characteristics

Variables	High GNRI (*n* = 241)	Low GNRI (*n* = 332)	*p*
**Age (years)**	70 ± 12	69 ± 10	0.631
**Sex**			0.956
Male, n (%)	114 (47.3%)	159 (47.9%)	
Female, n (%)	127 (52.7%)	173 (52.1%)	
**Diseases**,** n (%)**			
Hypertension	121 (50.2%)	157 (47.3%)	0.545
Dementia	27 (11.2%)	42 (12.7%)	0.692
Arthritis	81 (33.2%)	103 (31.0%)	0.573
Osteoporosis	46 (19.1%)	67 (20.2%)	0.827
Cancer	6 (2.5%)	11 (3.3%)	0.746
COPD	26 (10.8%)	35 (10.5%)	1.0
Diabetes	43 (17.8%)	67 (20.2%)	0.552
AKI	32 (13.3)	54 (16.3)	0.384
CKD	27 (11.2%)	41 (12.3%)	0.773
CLD	5 (2.1%)	8 (2.4%)	1.0
Stroke	11 (4.6%)	16 (4.8%)	1.0
Cardiovascular	182 (75.5%)	247 (74.4%)	0.835
**Weight (kg)**	67.37 ± 15.12	59.22 ± 12.10	0.004
**BMI (kg/m**^**2**^)	23.29 ± 4.69	20.82 ± 4.94	0.002
**NRS-2002 (score)**	2.93 ± 1.08	3.28 ± 0.79	0.150
**MNA-SF (score)**	8.98 ± 2.69	8.4 ± 2.29	0.350
**Lab test**			
ALB (g/L)	44.53 ± 5.54	32.98 ± 4.67	0.019
TP (g/L)	72.41 ± 8.13	61.21 ± 5.16	0.002
PA (mg/dl)	25.84 ± 4.23	17.05 ± 5.75	0.014
TRF (mg/dl)	232.67 ± 19.26	200.95 ± 20.27	0.415
RBP (mg/L)	28.02 ± 5.66	25.90 ± 4.22	0.098
FN (mg/L)	189.13 ± 25.81	198.46 ± 28.39	0.148
HGB (g/L)	120.04 ± 23.97	113.16 ± 20.65	0.217
RBC (10*^12^/L)	4.09 ± 0.63	3.89 ± 0.63	0.181
WBC (10*^9^/L)	7.53 ± 3.15	7.47 ± 3.11	0.936
LYM (10*^9^/L)	1.26 ± 0.61	1.46 ± 0.60	0.181
PRO (kg)	10.23 ± 8.66	6.86 ± 1.22	0.006
FAT (kg)	19.98 ± 8.77	23.66 ± 10.16	0.099
SM (kg)	24.98 ± 6.15	18.64 ± 3.65	< 0.001
BCM (kg)	29.39 ± 7.02	22.67 ± 4.01	< 0.001
VFA (cm^2^)	116.64 ± 37.19	138.69 ± 44.35	0.022
**HC (yuan)**	14186.79 ± 4013.00	21454.47 ± 7549.50	0.006
**LOS (day)**	10.40 ± 5.11	23.10 ± 5.49	0.002
**Hospital mortality**,** n (%)**	3 (1.2%)	7 (2.1%)	0.648
**3-month hospital readmission**	23 (9.5%)	56 (16.9%)	0.017
**6-month hospital readmission**	52 (21.6%)	104 (31.3%)	0.013

### Association of GNRI with hospital readmission

A Geriatric Nutritional Risk Index (GNRI) of 98 or higher is associated with a reduced chance of hospital readmission, both in univariate analysis and multivariate analysis (*p* < 0.001). In addition of GNRI, age also shows a significant correlation with readmission, particularly for those aged 80 or above. Gender does not exhibit a statistically significant association. For BMI, a value lower than 18.5 is linked with higher odds of higher chance of readmission. The NRS-2002 score of 3 or greater and MNA-SF scores below 11 also indicate higher risks for higher rate of hospital readmission in 6 months (Table 2).

**Table 2:** Univariate and multivariate logistic regression analyses of factors associated with hospital readmission

	Univariate		Multivariate	
	OR (95% CI)	*p*	OR (95%)	*p*
**GNRI**				
< 98	1		1	
≥ 98	0.63 (0.35–0.91)	< 0.001	0.55 (0.32–0.93)	< 0.001
**Age**				
60–69	1		1	
70–79	1.22 (1.01–1.54)	0.074		
≥ 80	1.43 (1.01–1.74)	0.046	1.52 (1.13–2.05)	0.028
**Gender**				
Male	1			
Female	0.94 (0.83–1.18)	0.483		
**BMI**				
18.5–24.9	1		1	
≤ 18.5	1.21 (0.78–1.52)	0.034	1.43 (1.05–1.94)	0.021
**NRS-2002**				
< 3	1		1	
≥ 3	1.75 (1.46–3.41)	< 0.001	1.82 (1.45–3.21)	< 0.01
**MNA-SF**				
> 11	1		1	
8–11	1.54 (1.03–1.95)	0.002	1.62 (1.16–2.01)	0.001
< 8	1.82 (1.23–2.43)	0.001	1.90 (1.34–2.74)	< 0.001

**Fig. 1:**
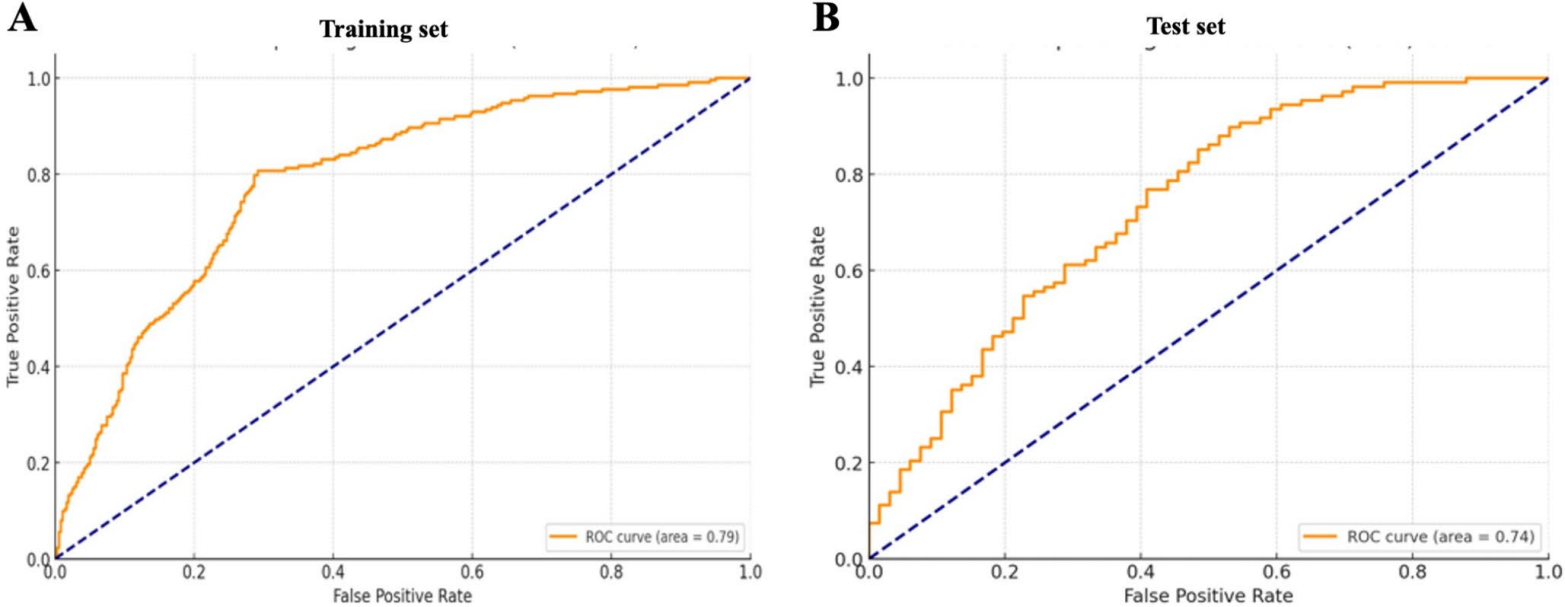
ROC analysis of GNRI for 6-month hospital readmission in the training set (**A**) (Sensitivity: 80.75%, Specificity: 70.83%. 95% CI: 0.76–0.81, *n* = 398) and validation set (**B**) (Sensitivity: 89.8% Specificity: 47.0% 95% CI: 0.67–0.80, *n* = 175)

To evaluate the sensitivity and specificity of the GNRI as a predictive tool for 6-month hospital readmission, we first randomly divided our participants into two subsets: the training set consists of approximately 70% (*n* = 398) of the total patient cohort and was utilized to identify the GNRI thresholds and refine the model parameters for maximum accuracy in predicting hospital readmissions, and the validation set which comprises the remaining 30% (*n* = 175) of the patient data and was not used in the model development phase. ROC analysis was performed in the training set (*n* = 398) and validation set (*n* = 175). The area under the ROC analysis with AUC = 0.79 (95% CI: 0.76–0.81) in the training set indicates a good predictive ability of GNRI for hospital readmission in 6 months of patients (Fig. 1A). The validate set also showed similar predictive ability (AUC = 0.74, 95% CI: 0.672–0.802) (Fig. 1B).

### Survival analysis and prognostic value of GNRI

**Fig. 2:**
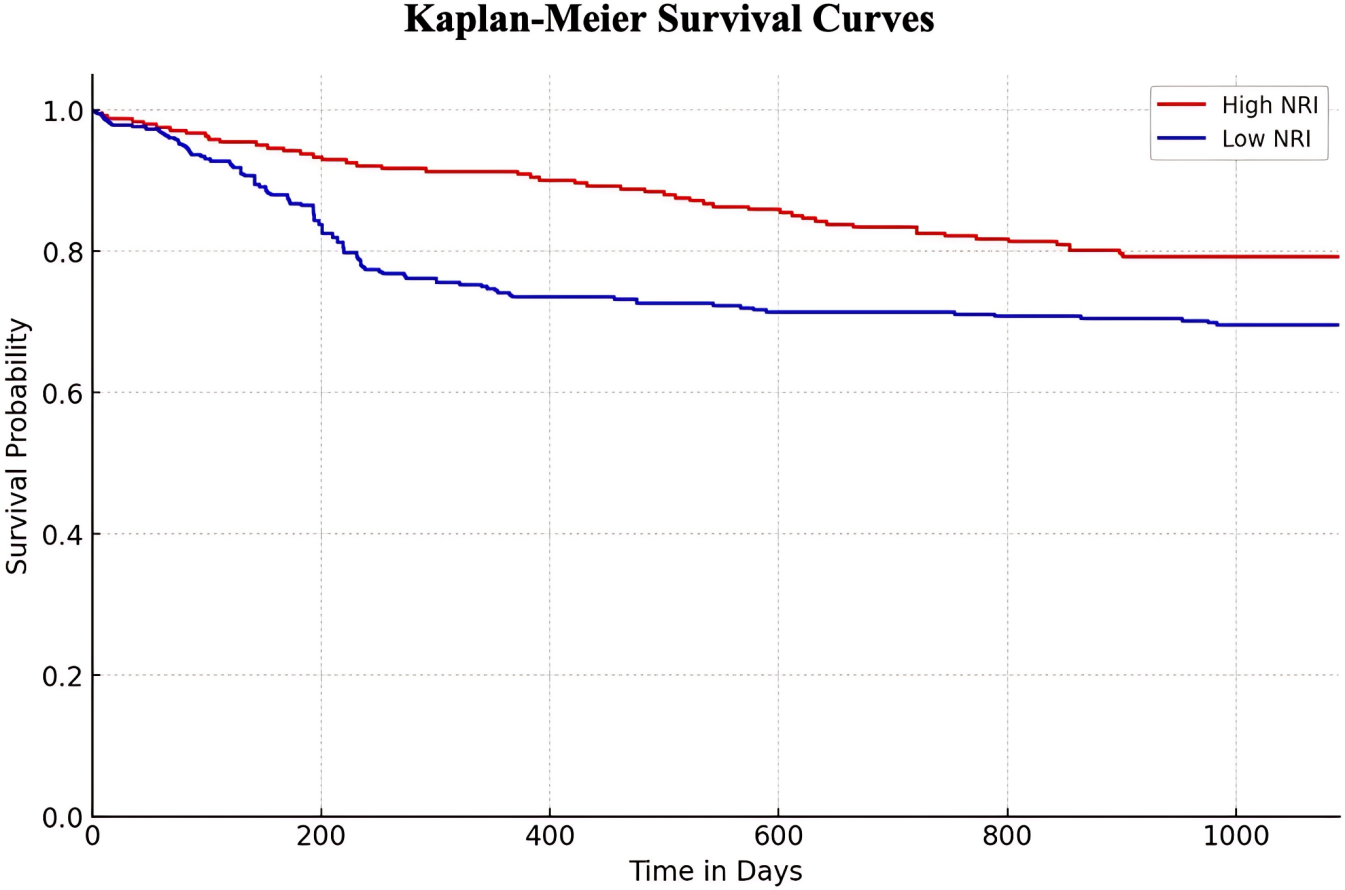
Kaplan-Meier analysis of 3-year survival in the High GNRI and Low GNRI group. Long-rank *p*-value: 0.0021

Three-year survival of participants with higher or low GNRI were performed to assess the association of survival probability with GNRI. Kaplan-Meier curve indicated that individuals with lower GNRI had significant decreased survival probability, compared with patients in the high GNRI (Fig. 2).

## Discussion

Our study investigated the relationship between the GNRI and clinical outcomes in older inpatients. We found that higher GNRI scores were significantly associated with improved clinical outcomes. Notably, patients categorized in the high GNRI group exhibited a statistically significant lower length of hospital stay and reduced hospital costs compared to the low GNRI group. Furthermore, these patients also demonstrated a lower rate of hospital readmission, underscoring GNRI’s potential as a valuable predictor of hospital readmission and overall hospital resource utilization.

The GNRI has been validated as a significant predictor of clinical outcomes in older patients, underscoring its utility in clinical settings to identify patients at nutritional risk and predict their clinical prognosis. Our study aligns with previous research indicating that a higher GNRI is associated with better nutritional status and lower risks of adverse outcomes such as prolonged hospital stays and increased mortality rates [10]. Similar to our results, Huang et al. demonstrated that a lower GNRI is predictive of worse outcomes and higher complication rates in older patients undergoing spinal tuberculosis surgery [11]. Additionally, Li et al. found that GNRI effectively predicted all-cause mortality in older patients with acute coronary syndrome over a 10-year period [10]. Moreover, a study reported that GNRI was a valuable indicator for predicting the length of hospital stay and mortality in older patients [12]. While it is well-documented that the GNRI is a validated tool for predicting adverse clinical outcomes, including mortality in various patient populations, previous studies have predominantly focused on GNRI’s utility in acute and single-disease contexts. Our study extends this understanding by examining GNRI’s prognostic value in a broad cohort of elderly inpatients with diverse medical backgrounds over a prolonged follow-up period. Our study specifically addresses the gap in longitudinal research exploring the utility of GNRI across a wide range of elderly inpatients, not limited to specific diseases or conditions. Prior studies have often been restricted by shorter follow-up durations or narrower patient demographics. We aimed to validate GNRI as a predictor of long-term outcomes, thus providing a comprehensive view of its predictive power across a diverse and aging hospital population.

Additionally, GNRI’s predictive power for mortality aligns with findings from other studies where it has been shown to predict all-cause mortality effectively, particularly in populations with acute medical conditions [13]. In our study, the relationship between GNRI and clinical outcomes was similarly profound, where higher GNRI scores correlated with lower rates of hospital readmission and improved survival rates. These observations are crucial for clinical practice, as they highlight the importance of integrating GNRI assessments into routine evaluations to better manage the older’s healthcare by tailoring interventions based on their nutritional risk.

This integrated approach to patient management could not only improve individual patient outcomes but also enhance the overall efficiency of healthcare systems by potentially reducing the frequency and length of hospitalizations [14]. As such, the GNRI serves not just as a clinical tool for assessing nutritional status but also as a broader indicator of patient resilience and recovery potential, which is particularly important in the geriatric population susceptible to multiple comorbidities and acute health fluctuations.

The efficacy of GNRI in predicting clinical outcomes is linked to its ability to highlight malnutrition, which is a known risk factor for poor recovery, higher complication rates, and increased mortality in older patients [15]. Malnutrition leads to diminished immune response, poorer wound healing, and reduced muscle strength, all of which contribute significantly to the morbidity and mortality in this population. Studies have demonstrated that improving nutritional status as reflected by GNRI scores can significantly reduce these risks, underscoring the importance of nutritional interventions in clinical settings [16].

Moreover, GNRI’s role extends beyond simple nutritional assessment to act as a surrogate marker for frailty and physiological reserve. Patients with higher GNRI scores typically have better physiological reserves, enabling them to withstand the stress of illness and medical interventions more effectively [17]. This relationship underscores why GNRI scores correlate strongly with outcomes such as length of hospital stay, postoperative complications, and overall mortality.

Our study benefits from a robust dataset spanning seven years, allowing us to track long-term outcomes in older patients. This longitudinal approach provides valuable insights into the sustained impacts of nutritional risk as assessed by GNRI on clinical outcomes such as hospital readmission and mortality. The inclusion of a broad demographic of older inpatients enhances the generalizability of our findings. This diversity ensures that the results are applicable to a wide range of clinical scenarios and not limited to specific conditions or treatments. By incorporating a variety of clinical and demographic variables in our analysis, including detailed body composition and various clinical scores, our study offers a comprehensive view of the factors influencing older patients’ health outcomes, highlighting the multifaceted nature of geriatric care. However, while these exclusions were necessary to maintain data consistency and reliability, they limit the applicability of our findings to all older inpatients. Patients with acute illnesses or severe psychiatric conditions might experience different nutritional risks and clinical outcomes, which are not captured in this study. In addition, the data were collected from only one hospital, there may be specific institutional practices that could influence the findings. Multi-center studies would be beneficial to confirm our results across different settings and patient management strategies. Moreover, the retrospective nature of our study, while facilitating the use of existing data, also imposes limitations on controlling for all potential confounders and biases inherent in historical records. Prospective studies would provide more control over data collection and the variables assessed. While GNRI is a proven tool for assessing nutritional risk, relying predominantly on this index may overlook other crucial factors affecting older patients’ health outcomes. Future studies could benefit from incorporating other nutritional and functional assessments to provide a more holistic view of the risks and needs of this population.

These consistent findings across different studies emphasize the reliability and validity of GNRI as a prognostic tool for assessing nutritional risk and predicting clinical outcomes in the older. These findings underscore the importance of nutritional status as a critical determinant in the recovery and health economics of older patients, suggesting that GNRI could serve as an effective tool for identifying patients at higher risk of adverse outcomes, thereby enabling timely and targeted nutritional interventions.

## Data Availability

The data that support the findings of this study are available from the corresponding author upon reasonable request.
